# Economic costs of asthma in Brazil: a one year real life study in outpatient setting

**DOI:** 10.1186/1939-4551-8-S1-A4

**Published:** 2015-04-08

**Authors:** Eduardo Costafsilva, Rogerio Rufino, Mauricio Bregman, Denizar Vianna Araújo, Claudia Costa

**Affiliations:** 1 State University of Rio De Janeiro, Brazil

## Background

Asthma cost have been studied predominantly in severe asthma and inpatient care. It is still unknown in different levels of severity and in different regions of the world. The aim was to estimate economic costs of asthma treatment in specialized outpatient care in a big city of Brazil.

## Methods

Persistent asthmatics ≥ 6 years old under GINA outpatient treatment were consecutively included in a real life design and a society perspective/bottom-up approach. They underwent routine clinical visits at 3- to 4-month intervals and 2 interviews with 6-month intervals using a structured instrument, made by researchers not involved in their clinical treatment. Data on asthma costs were collected directly from patients or parents, regarding prior 3 to 6 months . Collected data were valued in Brazilian Reais (R$) by using data banks from Brazillian Ministry of Health (for public resources), Brazillian Medical Association (for suplemmentary system resources) and values informed by patients for private resources expenditures. Exchange rate used was US$ 1.00 = R$ 1.89 (purchasing power parity in 2012/World Bank).

## Results

Of 117 subjects, 108 completed the study, with female predominance (n=79/73.8%). In initial evaluation, 16 (14.8%) had severe, 39 (36.2%) had moderate and 53 (49%) had mild asthma. Rhinitis was present in 83.3%, and 59.2% were overweighted or obese. The majority had elementary scholling (n=76/70.4%) and the mean monthly family income was US$ 1,202.90 (SD=880.37), the lowest stratum of medium economic class in Brazil. The estimated mean asthma cost was US$ 882.37 per patient-year (SD=717.97), corresponding to 7% of mean annual family income (MAFI) and to 6% of per capita gross domestic product (GDP). Estimated mean cost of asthma plus rhinitis and associated respiratory infections reached US$ 1,005.22 per patient-year (SD=724.07), which corresponds to 8.3% of MAFI and 7% of per capita GDP. The severe, not controlled, obese and overweight asthmatics had greater asthma costs when compared to mild, controlled and normal weight ones, (differences=449.5%, 142.7%, 51.7% and 35.6%, respectively).

## Conclusions

It is the first study describing data on associated costs of outpatient treatment of asthma in different severity levels in Brazil, which with think can be useful for public health policies. The annual estimated cost represents an important impact on family budgets and per capita GDP. Public health strategies offering wide access to treatment and stimulating weight reduction can contribute to better results, reducing costs of asthma in Brazil.

